# Trends in low-frequency underwater noise off the Oregon coast and impacts of COVID-19 pandemica)

**DOI:** 10.1121/10.0005192

**Published:** 2021-06-09

**Authors:** Peter H. Dahl, David R. Dall'Osto, Michael J. Harrington

**Affiliations:** Applied Physics Laboratory, University of Washington, Seattle, Washington 98105, USA

## Abstract

Approximately six years of underwater noise data recorded from the Regional Cabled Array network are examined to study long-term trends. The data originate from station HYS14 located 87 km offshore of Newport, OR. The results indicate that the third-octave band level centered at 63 Hz and attributable to shipping activity is reduced in the spring of 2020 by about 1.6 dB relative to the mean of the prior five years, owing to the reduced economic activity initiated by the COVID-19 pandemic. The results are subtle, as the noise reduction is less than the typical seasonal fluctuation associated with warming ocean surface temperatures in the summer that reduces mode excitation support at typical ship source depths, causing a repeated annual level change on the order of 4 dB at shipping frequencies. Seasonality of the noise contribution near 20 Hz from fin whales is also discussed. Corroboration of a COVID-19 effect on shipping noise is offered by an analysis of automatic identification system shipping data and shipping container activity for Puget Sound, over the same six-year period, which shows a reduction in the second quarter of 2020 by ∼19% and ∼17%, respectively, relative to the mean of the prior five years.

## I. INTRODUCTION

**FIG. 1.**
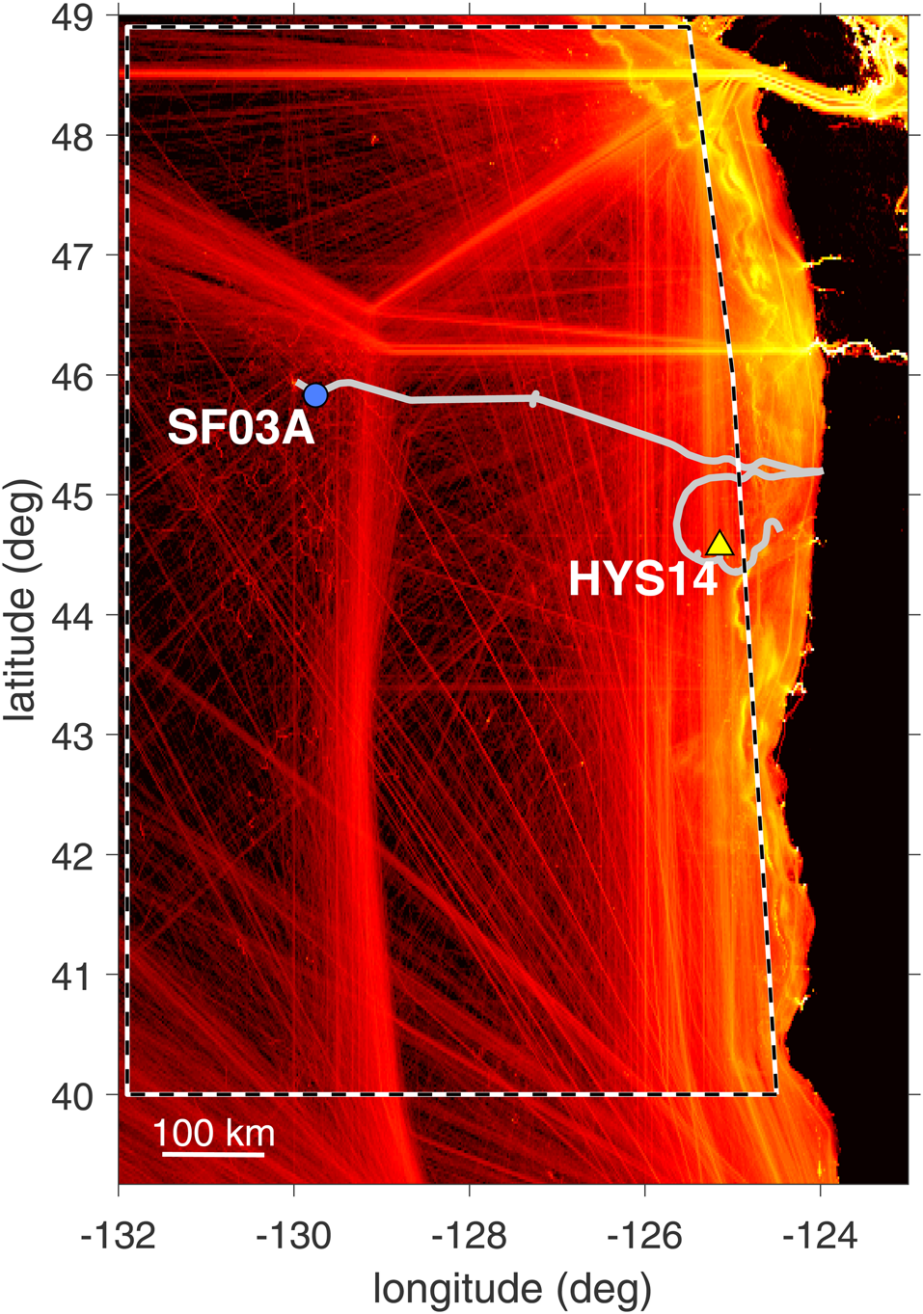
(Color online) Heat map of AIS reports in the Northeast Pacific Ocean 2015–2020, location of RCA cabling (gray lines), station HYS14 (triangle), and water column measurement site SF03A (circle). The white/black dashed outline identifies the area used to quantify shipping density.

The Ocean Observatories Initiative (OOI) Regional Cabled Array (RCA),^1,2^ funded by the National Science Foundation, provides a constant stream of data from the seafloor and water column spanning the Juan de Fuca plate. Here, we present underwater sound data recorded at the RCA site known as Hydrate Ridge station HYS14 (Fig. 1), located on the seabed at a depth of 778 m, approximately 87 km offshore of Newport, OR (44.569° N, 125.148° W). We examine long-term trends in shipping-related underwater noise and how trends compare to recent conditions that may have been impacted by the COVID-19 pandemic.

The HYS14 hydrophone commenced data streaming on 15 January 2015, and this study incorporates all data from that point until 17 August 2020, after which the primary node (PN1B) of the RCA developed a fault that took part of the RCA infrastructure offline. (Replacement of the failed node is scheduled for summer 2021.) Station HYS14 was selected for analysis primarily because there are fewer noise producing real-time measurement systems at this station relative to other stations within the RCA system. The site is well situated within north-south coastal shipping lanes (Fig. 1) and likely to experience more distant traffic noise^3,4^ associated with great-circle shipping routes connecting northwest ports (e.g., Vancouver, BC, and Seattle and Tacoma, WA) with Asian ports.

The paper is organized as follows: Sec. II describes data and processing methods in the frequency domain. Note that we use both linear and geometric averages to describe measures of central tendency in spectral and mean-square levels depending on the purpose, which will be explained. Section III provides results, including a short summary of the biophony that must be understood in the context of the noise related to shipping. Here, we also document the seasonal propagation effect for near-surface radiated shipping noise, including simple modeling that captures the trends. These two elements provide necessary background for our conclusions related to underwater noise shipping and economic impacts of COVID-19, discussed in Sec. IV and summarized in Sec. V.

## II. DATA DESCRIPTION AND PROCESSING METHODS

The data were obtained through the Incorporated Research Institutions for Seismology (IRIS) network, accessing station OO, sensor HYS14. The sampling frequency is 200 Hz applied after data are low-pass filtered with a 3-dB corner frequency of about 90 Hz prior to sampling. The HYS14 hydrophone is an HTI-90-U low-frequency hydrophone manufactured by HiTech (Long Beach, MS), with effective receiving sensitivity (inclusion of gain) equal to –174 dB re V/μPa.

The basic spectral analysis revolves around the sequence of whole hours for a given day as recorded in UTC, as follows. We take the fast Fourier transform (FFT) of Hanning tapered blocks equal to 12 000 points ( T= 60 s) and average 119 adjacent blocks overlapped by 50%, corresponding to a 1-h spectral estimate following Welch's overlapped segment average (e.g., see Percival^5^). These one-sided variance spectra $S_{n}(f)$ integrate to the mean-square pressure of the corresponding *n*th-hour non-tapered block. For a given day, the pressure variance spectrum $P_{ms}(f)=\langle S_{n}(f)\rangle$, in dimension Pa^2^/Hz; we also compute $P_{dB}(f)$ equal to $\left\langle 10\;{\text{log}}_{10}S_{n}(f)\right\rangle$, representing a geometric mean over the day. The geometric mean is sensitive to the time scale of the spectral estimate, and after some experimentation, 1 h, as also suggested in Ref. 6, provides the suitable balance that captures longer-term seasonal trends and mitigates the influence of transient events.

Two correlates to the acoustic data are based on direct measurements of the sound speed profile and shipping density. Sound speed is derived from measurements at RCA station SF03A, located 388 km from the HYS14 site (Fig. 1); here a winched shallow-water profiler continuously measures conductivity and temperature over the upper 200 m.

Shipping density is assessed by automatic identification system (AIS) reports^7^ distilled into a count of the number of ships with unique International Maritime Organization (IMO) numbers, limited in this study to ships >100 m length within ∼600 km west, north, or south of HYS14 (within the white/black dashed line in Fig. 1).

## III. RESULTS

**FIG. 2.**
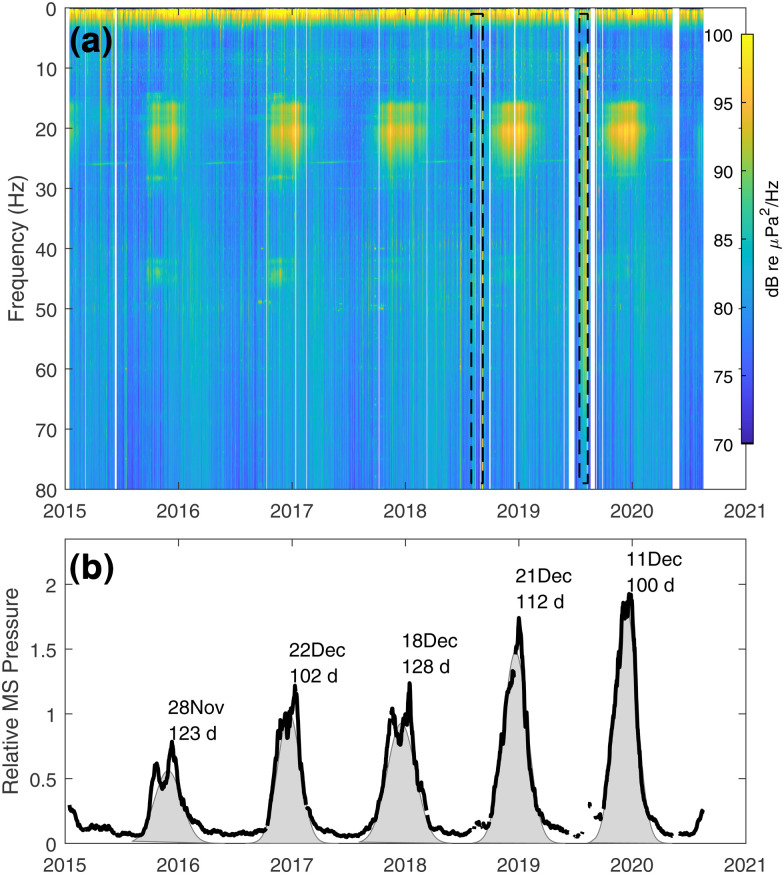
(Color online) (a) Daily averaged $P_{dB}(f)$ spanning the period 15 January 2015-17 August 2020 for RCA hydrophone HYS14. Black, dashed rectangles identify research vessel activity in 2018 and a seismic survey in 2019. (b) Mean-square pressure as computed from daily averaged $P_{ms}(f)$ integrated over the 18.5–22.5 Hz band and subjected to a 30-day moving geometric average. The mean-square pressures are normalized in the plot such that 1 is mapped to a mean-square pressure equivalent to 100 dB re μPa^2^, and 2 is equivalent to 103 dB. A separate Gaussian fit is made for each year (shaded curves) with first moment date and 2*σ* spread in days annotated.

A summary of the entire HYS14 hydrophone data set to date is displayed in the stacked image of the daily $P_{dB}(f)$ spanning the period 15 January 2015–17 August 2020 [Fig. 2(a)]. Occasional data interruptions amount to 83 missing days during this period of 5 years and 7 months. Black, dashed rectangles identify known research vessel activity occurring above the sensor (2018) and a nearby seismic survey (2019), which are removed from subsequent analysis.

A prominent seasonal feature of the data is seen in the band centered near 20 Hz, which is consistent with that produced by fin whales (*Balaenoptera physalus*).^8,9^ Examining select periods (typically hours in duration) while this signal is present, a representative coherence time scale *τ_e_*, e.g., as defined in Ref. 10, is ∼0.25 s, with center frequency ∼20.5 Hz. Less prominent is a higher-frequency counterpart, between 40 and 45 Hz; this has been attributed to feeding calls by fin whales,^11^ although generally not as contemporaneous with 20-Hz calls as observed here.

For the 20-Hz signal, it is of interest to take the daily average of $P_{ms}(f)$, integrated over the $1/\tau_{e}$ or the 4-Hz band, 18.5–22.5 Hz, and apply a 30-day moving (geometric) average using the decibel equivalent. Converting back to mean-square pressure, the results agree with Gaussian fits made for each year to describe behavioral timing, with the date of the first moment falling between 28 November and 22 December and 2*σ* spread ranging from 100 to 128 days [Fig. 2(b)]. To assess variation, the pre-averaged data are detrended by the moving-average result, yielding an approximately Gaussian variation that is expected^10^ with standard deviation ∼2.5 dB, most applicable in the vicinity of the period of each first moment date.

The driver of this timing, for example, as in migration or enhanced call activity during breeding periods, is beyond the scope of this study, although it is noteworthy that the mean-square pressure in this band generally increases with advancing year, whereas the 2*σ* duration in days times mean-square pressure remains approximately constant. We also observe a narrow band tone at ∼26 Hz during the minima in fin whale calls, suspected to be a chorus of blue whale (*Balaenoptera musculus*) calls. While individual calls are not discernible in these data, the frequency of the tone modulates with the season and decreases at a rate of 1/8 Hz per year, consistent with other observations in the Southern Hemisphere.^12,13^

These are rich and stand-alone investigations that are important to mention here for context. Our main focus in this study, however, is on noise from shipping, for which we limit our analysis to the third-octave (decidecadal) band spanning 56.23–70.79 Hz centered at 63.10 Hz to be sufficiently separated in frequency from this biophony. This corresponds, for example, to one of the important third-octave bands studied for long-term trends from shipping noise along the U.S. West Coast between 1996 and 2007.^4^

The daily $P_{dB}(f)$ is further averaged over this band, as this is the most effective method to characterize the seasonal pattern in the band spectral level that is linked to annual ocean surface temperature cycles, as reported by others.^14^ The geometric average, similar to taking the median, tends to stabilize the variance and reduces the influence of episodic high outlying values. Unlike the median, all values contribute to the average.

**FIG. 3.**
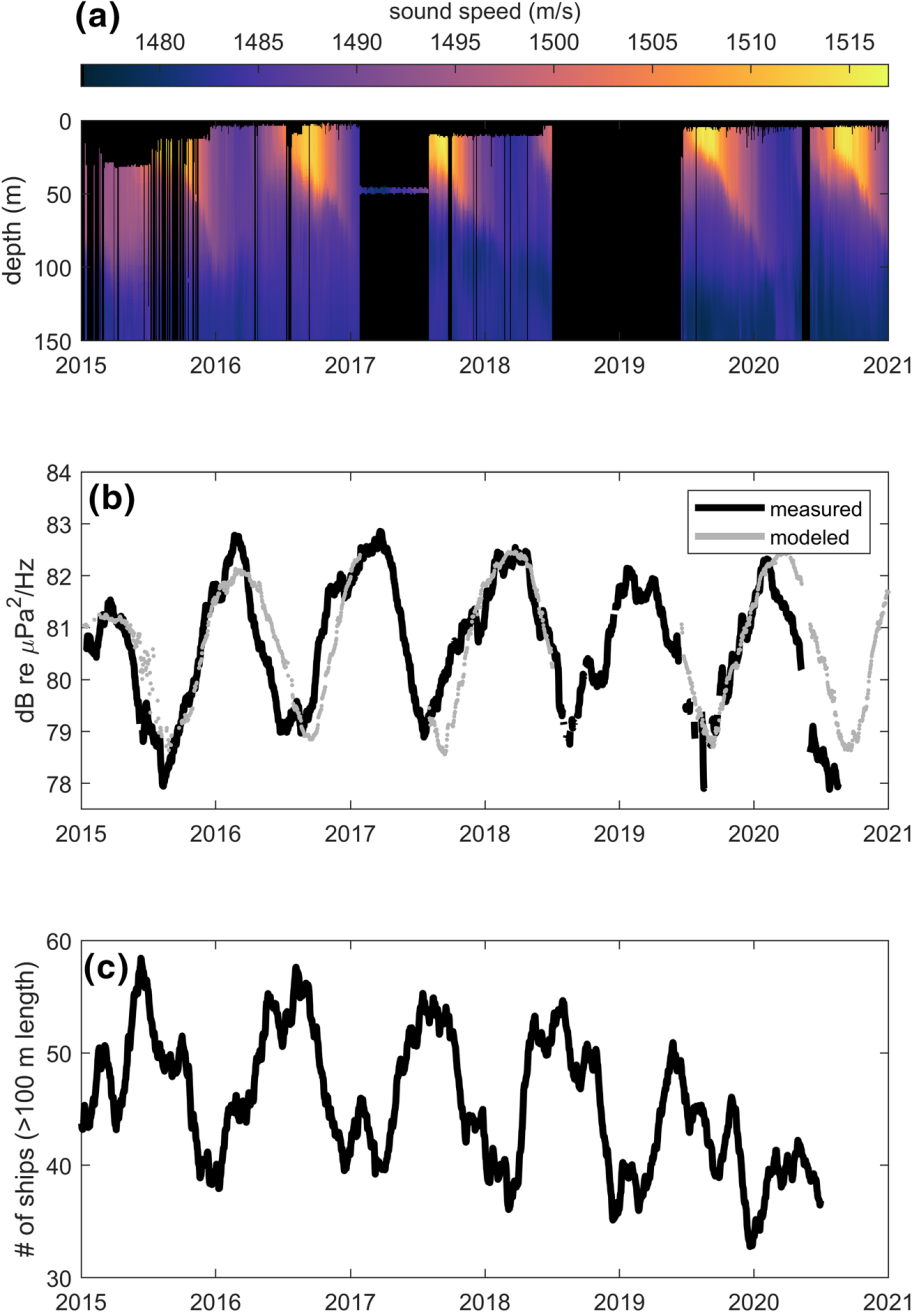
(Color online) (a) Sound speed profiles measured at SF03A; black regions indicate no data. (b) Average $P_{dB}(f)$ within the 63-Hz third-octave band (black line) smoothed by a 45-day moving average and modeled levels (gray line) based on the measured sound speed profile. (c) A 45-day moving average of number of ships with length >100 m passing within the region indicated in Fig. 1, as identifed by unique IMO number.

With seasonal warming and onset of higher ocean surface temperatures, it is expected that mode excitation support moves deeper and thus away from typical ship source depths. Evidence for this comes from converting the shallow-water profiler data from RCA station SF03A to sound speed [Fig. 3(a)], which can be used in simple modeling. A 45-day moving average of $P_{dB}(f)$ (intentionally longer than in the case of the fin whale data) is taken [black line, Fig. 3(b)], which shows a remarkable seasonal trend, and detrending yields a Gaussian variation with standard deviation ∼2 dB. This trend is reasonably captured upon using the sound speed data [Fig. 3(a)]^15^ and computing an incoherent sum of modes based on mode excitation coefficients for a 60-Hz source at a notional source depth of 5 m multiplied by the respective propagation decay (imaginary part of wavenumber). The results are summed for a uniform distribution of sources from 1 to 600 km, yielding a model (not calibrated) of the seasonal pattern in band spectral level with variation [gray line in Fig. 3(b)] that is in phase with the long-term observations of shipping noise. Wind-related noise will have some influence, but less at frequencies lower than about 100 Hz.^16^ However, it is also not plausible for it to be manifested in such a repetitive manner over the course of six annual cycles; furthermore, wind speeds at this site are relatively constant during the year.

Referring to this now as a seasonal propagation effect for near-surface generated shipping noise, it is also evident that ship density as reflected by the daily count of vessels over 100-m length within the box in Fig. 1, is *out of phase* with the seasonal propagation effect, i.e., highest shipping activity generally occurs in mid-year [Fig. 3(c)].

These results (Fig. 3) suggest that any underlying trend in the underwater noise data associated with COVID-19 will necessarily be subtle. To examine this trend, we return to the daily (linear) $P_{ms}(f)$, taking the average over the 63-Hz third-octave band. Operating with daily $P_{ms}(f)$, as with the fin whale case (but here maintaining units of spectral density), is more appropriate given our goal to assess the addition of incoherent sources^17^ rather than a seasonal long-term propagation trend (Fig. 3), which uses $P_{dB}(f)$.

**FIG. 4.**
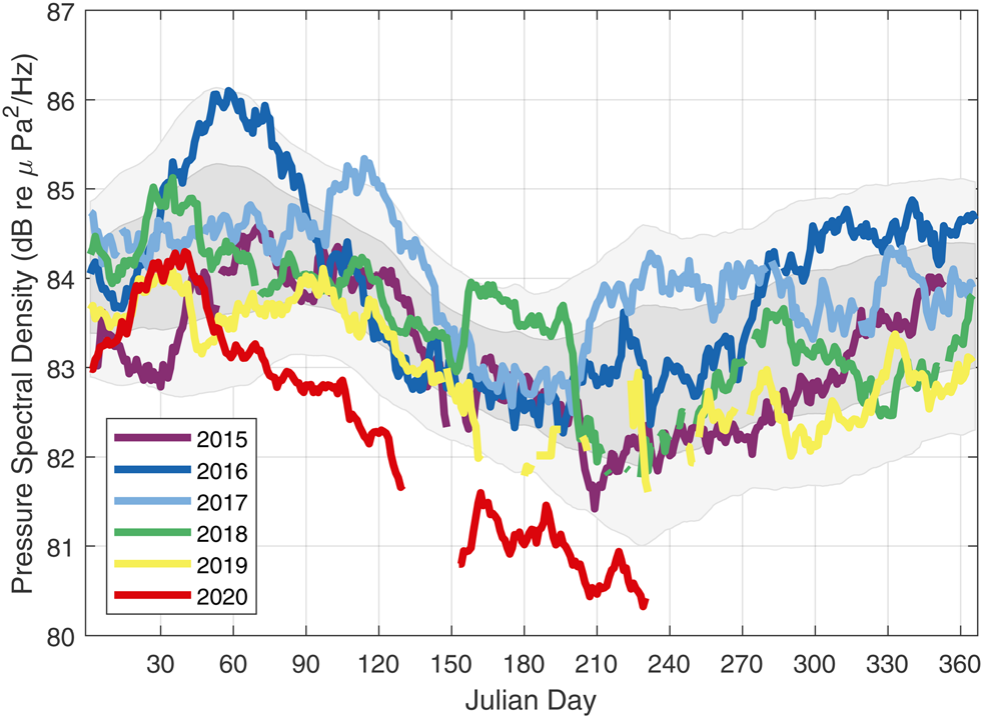
(Color online) A 45-day moving geometric average of the 63-Hz third-octave band levels for years 2015–2020 (system offline between days 120 and 150 in 2020). Gray shaded areas denote ±1*σ* and 2*σ* about this mean trend based on grouping the data over the first five years and computing the mean and standard deviation over time.

The 45-day moving geometric average of the 63-Hz band level is plotted with respect to Julian day for each year (Fig. 4). The seasonal dependence [Fig. 3(b)] is evident, although reduced when based on $P_{ms}(f)$ with a peak-to-trough difference of about 2–3 dB, with the 63-Hz band levels over the six years reaching an annual minimum near day 210.

It is also apparent that the level for year 2020 departs from the previous five years starting at around day 60. The departure is not large but is persistent, starting at about ∼1 dB and increasing to ∼2 dB. This result is notionally consistent with recent results obtained from the Canadian network north of our study site.^18^ Grouping the data from the prior five years and computing the mean and standard deviation over time, the gray shaded areas denote ±1*σ* and 2*σ* about this mean trend; after about day 60, year 2020 remains less than this mean trend by at least 2*σ*.

## IV. DISCUSSION

The duration over which to compare the influence of suppressed economic activity due to the COVID-19 pandemic on underwater noise levels with prior years is admittedly brief, but the second economic quarter (Q2) of each year provides some corroborating evidence. The Q2 quarterly averages (Table I) of the 63-Hz band levels (Fig. 4) put Q2 of 2020 about 1.6 dB less than the mean of Q2 for the prior five years. Similarly, the 2020 average daily large-vessel count for Q2 (Table I) is reduced by ∼19% upon comparing with the mean of Q2 for the prior five years. It is plausible to assume that noise goes as $10\;{\text{log}}_{10}$ of the large-vessel count, with the ∼19% reduction yielding a change of about ∼1 dB, which is nominally consistent. Furthermore, the 2020 Q2 TEU totals for Puget Sound are reduced by ∼17%. It has been proposed^19^ that low-frequency shipping noise levels scale with gross tonnage, and possibly this may be a better predictor.

**TABLE I.** Metrics for Q2 (April–June).

Year	63-Hz band dB re 1 μPa^2^/Hz	Quarterly TEU^a^	Traffic (number of vessels)^b^
2015	83.5	925 735	51
2016	83.3	895 732	52
2017	84.1	929 716	47
2018	83.7	940 241	49
2019	83.1	982 961	47
2020	81.9	775 382	40

^a^ Twenty-foot equivalent unit (TEU).

^b^ Average daily count of vessels length >100 m, passing within the area indicated in Fig. 1.

Note that the estimated reduction of 1.6 dB is less than, for example, the relative reduction in noise levels observed in the rerouting of shipping lanes away from the measurement site, as discussed in the context of the 2008 economic downturn,^20^ although it does not appear that rerouting is in effect in our study. Nevertheless, our findings over this shorter period of economic change should be viewed as more substantive in quantifying the reduction in noise level and less so in unraveling the precise causative association with measures of shipping activity.

## V. SUMMARY

Underwater noise data measured 87 km off Newport, OR between 15 January 2015 and 17 August 2020 are examined by comparing the 2020 data with that from the prior five years. We find a 1.6-dB reduction in the 63-Hz third-octave band spectral level that is typically associated with underwater noise from shipping. The reduction begins on approximately Julian day 60 of 2020 and is relative to the same period in the prior five years, which we infer is the result of reduced shipping and economic activity impacted by the COVID-19 pandemic. Corroboration of this COVID-19 effect is offered through an analysis of AIS shipping data and shipping container activity for Puget Sound. Our results are subtle and must be understood in the context of the significant seasonality within this band on the order of 4 dB, which is in phase with seasonal changes in Northeast Pacific Ocean temperature in the upper 200 m. The seasonally dependent minimum in noise level is concurrent with the reduced shipping activity during the COVID-19 pandemic.
